# 2,2′,2′′-[Nitrilo­tris(methyl­ene-*p*-phenyl­ene)]tribenzonitrile

**DOI:** 10.1107/S1600536808029784

**Published:** 2008-09-20

**Authors:** Li-Zhuang Chen

**Affiliations:** a Ordered Matter Science Research Center, College of Chemistry and Chemical Engineering, Southeast University, Nanjing 210096, People’s Republic of China

## Abstract

In the title compound, C_42_H_30_N_4_, the conformations of the three wings of the mol­ecule are not similar to each other as the torsion angles between the planes of the benzene rings are significantly different. In addition to van der Waals inter­actions, the crystal structure is stabilized only by intra­molecular C—H⋯N hydrogen bonds.

## Related literature

For related structures, see: Fox *et al.* (1996 ▶); Menage *et al.* (1992 ▶); Murthy & Karlin (1993 ▶); Schrock (1997 ▶); Foces-Foces *et al.* (1999 ▶); Chen *et al.* (2005 ▶); Iwasaki & Iwasaki (1972 ▶).
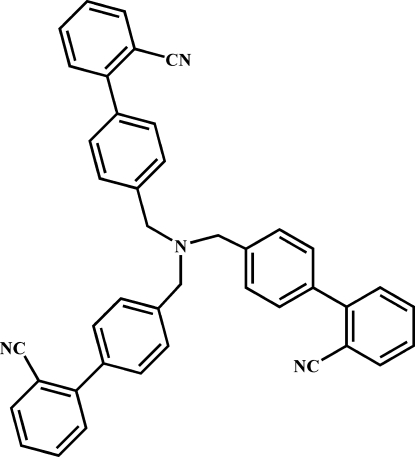


## Experimental

### 

#### Crystal data

C_42_H_30_N_4_
*M* *_r_* = 590.70
Monoclinic, 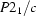

*a* = 21.257 (4) Å
*b* = 15.085 (3) Å
*c* = 10.294 (2) Å
β = 94.73 (3)°
*V* = 3289.5 (11) Å^3^
*Z* = 4
Mo *K*α radiation
μ = 0.07 mm^−1^
*T* = 298 (2) K
0.27 × 0.18 × 0.15 mm

#### Data collection

Rigaku Mercury2 diffractometer
Absorption correction: multi-scan (*CrystalClear*; Rigaku, 2005 ▶) *T*_min_ = 0.985, *T*_max_ = 0.989
32746 measured reflections
7524 independent reflections
4007 reflections with *I* > 2σ(*I*)
*R*_int_ = 0.081

#### Refinement

*R*[*F* ^2^ > 2σ(*F* ^2^)] = 0.065
*wR*(*F* ^2^) = 0.187
*S* = 1.02
7524 reflections
415 parameters
6 restraints
H-atom parameters constrained
Δρ_max_ = 0.17 e Å^−3^
Δρ_min_ = −0.24 e Å^−3^

### 

Data collection: *CrystalClear* (Rigaku, 2005 ▶); cell refinement: *CrystalClear*; data reduction: *CrystalClear*; program(s) used to solve structure: *SHELXS97* (Sheldrick, 2008 ▶); program(s) used to refine structure: *SHELXL97* (Sheldrick, 2008 ▶); molecular graphics: *SHELXTL* (Sheldrick, 2008 ▶); software used to prepare material for publication: *SHELXL97*.

## Supplementary Material

Crystal structure: contains datablocks I, global. DOI: 10.1107/S1600536808029784/pv2103sup1.cif

Structure factors: contains datablocks I. DOI: 10.1107/S1600536808029784/pv2103Isup2.hkl

Additional supplementary materials:  crystallographic information; 3D view; checkCIF report

## Figures and Tables

**Table 1:** Hydrogen-bond geometry (Å, °)

*D*—H⋯*A*	*D*—H	H⋯*A*	*D*⋯*A*	*D*—H⋯*A*
C4—H4*A*⋯N3^i^	0.93	2.55	3.446 (5)	162
C24—H24*A*⋯N2^ii^	0.93	2.59	3.350 (4)	139

Symmetry codes: (i) 
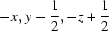
; (ii) 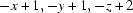.

**Fig. 1.**
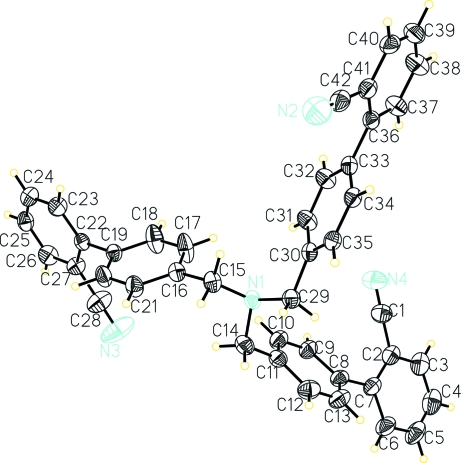
A view of the title compound with the atomic numbering scheme. Displacement ellipsoids were drawn at the 30% probability level.

## supplementary crystallographic information

### Comment

Tripodal ligands have shown tremendous scope in the synthesis of transition-metal complexes. Perhaps the most extensively studied examples are those comprising tris-(2-pyridylmethyl)amine, which has been widely exploited in complexes (Fox *et al.* 1996; Menage *et al.* 1992; Murthy & Karlin 1993), tris(2-aminobenzyl)amine (Foces-Foces *et al.* 1999), tris(2-chlorobenzl)amine and tris(2-bromobenzyl)amine (Chen *et al. *2005) and tribenzylamine (Iwasaki & Iwasaki 1972). The construction of new members of this family of ligands is an important direction in the development of modern coordination chemistry (Schrock 1997). We report here the crystal structure of the title compound tris[4-(2-cyano-phenyl)benzyl]amine, (I).

In the title compound (Fig.1), the conformations of the three wings of the molecule do not appear to be similar as the torsion angles between the planes of benzene rings are significantly different from each other. The pairs of benzene rings in the three wings form dihedral angle of 47.70 (10)°, 54.36 (7)° and 87.89 (9)°, respectively. In addition to van der Waals interactions, the crystal structure is stabilized only by intramolecular C—H···N hydrogen bonds: C4—H4A···N3 and C24—H24A···N2 (Table 1).

### Experimental

The title compount was obtained from the combination of 2-cyano-4'-(bromomethyl)biphenyl (8.13 g, 30.0 mmol) with 29% aqueous ammonia (2.64 g, 45.0 mmol) in ethanol (50 ml) at room temperature. A white solid precipitated during stirring over 24 h. The precipitate was collected by filtration, washed with ethanol, and allowed to air-dry to yield 2.67 g (43%) of colorless microcrystals. Recrystallization was effected from hot acetonitrile to yield colorless blocks suitable for X-ray analysis.

### Refinement

All H atoms were fixed geometrically and treated as riding with C–H = 0.93 Å(aromatic), 0.97 Å(methylene), and *U*_iso_(H) = 1.2Ueq(C).

### Crystal data

**Table:**

C_42_H_30_N_4_	*F*(000) = 1240
*M**_r_* = 590.70	*D*_x_ = 1.193 Mg m^−^^3^
Monoclinic, *P*2_1_/*c*	Mo *K*α radiation, λ = 0.71073 Å
Hall symbol: -P 2ybc	Cell parameters from 7512 reflections
*a* = 21.257 (4) Å	θ = 3.2–27.5°
*b* = 15.085 (3) Å	µ = 0.07 mm^−^^1^
*c* = 10.294 (2) Å	*T* = 298 K
β = 94.73 (3)°	Block, colorless
*V* = 3289.5 (11) Å^3^	0.27 × 0.18 × 0.15 mm
*Z* = 4	

### Data collection

**Table:**

Rigaku Mercury2 (2x2 bin mode) diffractometer	7524 independent reflections
Radiation source: fine-focus sealed tube	4007 reflections with *I* > 2σ(*I*)
graphite	*R*_int_ = 0.081
Detector resolution: 13.6612 pixels mm^-1^	θ_max_ = 27.5°, θ_min_ = 3.2°
ω scans	*h* = −27→27
Absorption correction: multi-scan (CrystalClear; Rigaku, 2005)	*k* = −19→19
*T*_min_ = 0.985, *T*_max_ = 0.989	*l* = −13→13
32746 measured reflections	

### Refinement

**Table:**

Refinement on *F*^2^	Primary atom site location: structure-invariant direct methods
Least-squares matrix: full	Secondary atom site location: difference Fourier map
*R*[*F*^2^ > 2σ(*F*^2^)] = 0.065	Hydrogen site location: inferred from neighbouring sites
*wR*(*F*^2^) = 0.187	H-atom parameters constrained
*S* = 1.02	*w* = 1/[σ^2^(*F*_o_^2^) + (0.0804*P*)^2^ + 0.198*P*] where *P* = (*F*_o_^2^ + 2*F*_c_^2^)/3
7524 reflections	(Δ/σ)_max_ < 0.001
415 parameters	Δρ_max_ = 0.17 e Å^−^^3^
6 restraints	Δρ_min_ = −0.24 e Å^−^^3^

### Special details

**Table:**

Geometry. All e.s.d.'s (except the e.s.d. in the dihedral angle between two l.s. planes) are estimated using the full covariance matrix. The cell e.s.d.'s are taken into account individually in the estimation of e.s.d.'s in distances, angles and torsion angles; correlations between e.s.d.'s in cell parameters are only used when they are defined by crystal symmetry. An approximate (isotropic) treatment of cell e.s.d.'s is used for estimating e.s.d.'s involving l.s. planes.
Refinement. Refinement of *F*^2^ against ALL reflections. The weighted *R*-factor *wR* and goodness of fit *S* are based on *F*^2^, conventional *R*-factors *R* are based on *F*, with *F* set to zero for negative *F*^2^. The threshold expression of *F*^2^ > σ(*F*^2^) is used only for calculating *R*-factors(gt) *etc*. and is not relevant to the choice of reflections for refinement. *R*-factors based on *F*^2^ are statistically about twice as large as those based on *F*, and *R*- factors based on ALL data will be even larger.

### Fractional atomic coordinates and isotropic or equivalent isotropic displacement parameters (Å^2^)

**Table:**

	*x*	*y*	*z*	*U*_iso_*/*U*_eq_	
C1	0.10835 (14)	0.41593 (18)	0.1399 (3)	0.0704 (7)	
C2	0.04158 (12)	0.40234 (15)	0.1365 (3)	0.0635 (7)	
C3	0.00882 (16)	0.39289 (18)	0.0152 (3)	0.0819 (9)	
H3A	0.0302	0.3986	−0.0596	0.098*	
C4	−0.05428 (19)	0.3753 (2)	0.0036 (4)	0.0946 (11)	
H4A	−0.0759	0.3692	−0.0781	0.114*	
C5	−0.08530 (15)	0.3669 (2)	0.1143 (4)	0.0947 (11)	
H5A	−0.1283	0.3546	0.1068	0.114*	
C6	−0.05411 (12)	0.37614 (18)	0.2376 (3)	0.0818 (9)	
H6A	−0.0763	0.3695	0.3113	0.098*	
C7	0.01046 (11)	0.39535 (14)	0.2513 (3)	0.0604 (7)	
C8	0.04395 (10)	0.40436 (15)	0.3830 (3)	0.0576 (6)	
C9	0.08923 (11)	0.46989 (15)	0.4118 (3)	0.0613 (7)	
H9A	0.0968	0.5120	0.3489	0.074*	
C10	0.12276 (11)	0.47328 (16)	0.5310 (3)	0.0623 (7)	
H10A	0.1529	0.5175	0.5470	0.075*	
C11	0.11309 (11)	0.41278 (15)	0.6284 (2)	0.0559 (6)	
C12	0.06633 (12)	0.35033 (17)	0.6025 (3)	0.0706 (7)	
H12A	0.0572	0.3105	0.6673	0.085*	
C13	0.03285 (12)	0.34602 (17)	0.4821 (3)	0.0720 (8)	
H13A	0.0020	0.3027	0.4671	0.086*	
C14	0.15284 (11)	0.41551 (17)	0.7558 (2)	0.0621 (7)	
H14A	0.1525	0.4752	0.7909	0.074*	
H14B	0.1348	0.3762	0.8174	0.074*	
C15	0.25982 (12)	0.41470 (14)	0.8554 (2)	0.0545 (6)	
H15A	0.3000	0.3845	0.8533	0.065*	
H15B	0.2409	0.3958	0.9334	0.065*	
C16	0.27119 (10)	0.51310 (14)	0.8625 (2)	0.0499 (6)	
C17	0.29125 (16)	0.55906 (17)	0.7584 (2)	0.0854 (9)	
H17A	0.2969	0.5288	0.6815	0.103*	
C18	0.30323 (16)	0.64887 (17)	0.7647 (2)	0.0843 (9)	
H18A	0.3166	0.6781	0.6923	0.101*	
C19	0.29559 (10)	0.69558 (14)	0.8768 (2)	0.0471 (5)	
C20	0.27620 (13)	0.64986 (16)	0.9803 (2)	0.0674 (7)	
H20A	0.2710	0.6800	1.0575	0.081*	
C21	0.26400 (12)	0.56002 (16)	0.9737 (2)	0.0641 (7)	
H21A	0.2507	0.5310	1.0463	0.077*	
C22	0.31100 (10)	0.79189 (13)	0.8858 (2)	0.0458 (5)	
C23	0.37045 (12)	0.82099 (15)	0.9309 (2)	0.0615 (7)	
H23A	0.4012	0.7794	0.9571	0.074*	
C24	0.38549 (13)	0.90998 (16)	0.9382 (2)	0.0641 (7)	
H24A	0.4260	0.9275	0.9682	0.077*	
C25	0.34097 (13)	0.97222 (16)	0.9014 (2)	0.0601 (6)	
H25A	0.3511	1.0322	0.9065	0.072*	
C26	0.28152 (13)	0.94626 (15)	0.8570 (3)	0.0657 (7)	
H26A	0.2512	0.9886	0.8318	0.079*	
C27	0.26623 (11)	0.85714 (15)	0.8493 (2)	0.0556 (6)	
C28	0.20387 (15)	0.83188 (18)	0.8029 (3)	0.0873 (10)	
C29	0.22125 (11)	0.29298 (14)	0.7186 (2)	0.0548 (6)	
H29A	0.1867	0.2761	0.6561	0.066*	
H29B	0.2154	0.2627	0.7998	0.066*	
C30	0.28224 (10)	0.26225 (13)	0.6692 (2)	0.0456 (5)	
C31	0.31594 (11)	0.31379 (14)	0.5885 (2)	0.0570 (6)	
H31A	0.3017	0.3707	0.5672	0.068*	
C32	0.37013 (11)	0.28319 (15)	0.5387 (2)	0.0572 (6)	
H32A	0.3920	0.3197	0.4852	0.069*	
C33	0.39231 (10)	0.19792 (14)	0.5679 (2)	0.0455 (5)	
C34	0.35817 (10)	0.14595 (13)	0.6485 (2)	0.0472 (5)	
H34A	0.3717	0.0886	0.6689	0.057*	
C35	0.30462 (10)	0.17814 (13)	0.6985 (2)	0.0473 (5)	
H35A	0.2830	0.1423	0.7534	0.057*	
C36	0.44926 (10)	0.16442 (13)	0.5103 (2)	0.0480 (5)	
C37	0.45515 (12)	0.17505 (16)	0.3787 (3)	0.0653 (7)	
H37A	0.4230	0.2033	0.3276	0.078*	
C38	0.50730 (14)	0.14503 (19)	0.3209 (3)	0.0791 (8)	
H38A	0.5099	0.1531	0.2319	0.095*	
C39	0.55515 (13)	0.10347 (17)	0.3934 (3)	0.0745 (8)	
H39A	0.5903	0.0836	0.3539	0.089*	
C40	0.55158 (11)	0.09101 (15)	0.5233 (3)	0.0654 (7)	
H40A	0.5842	0.0625	0.5726	0.078*	
C41	0.49847 (11)	0.12139 (14)	0.5828 (2)	0.0568 (5)	
C42	0.49593 (11)	0.10864 (16)	0.7198 (3)	0.0615 (5)	
N1	0.21846 (8)	0.38865 (11)	0.74051 (18)	0.0498 (5)	
N2	0.49418 (13)	0.09735 (19)	0.8305 (3)	0.1000 (9)	
N3	0.15370 (15)	0.8150 (2)	0.7631 (4)	0.1453 (15)	
N4	0.16140 (12)	0.42632 (19)	0.1407 (3)	0.0936 (8)	

### Atomic displacement parameters (Å^2^)

**Table:**

	*U*^11^	*U*^22^	*U*^33^	*U*^12^	*U*^13^	*U*^23^
C1	0.0622 (18)	0.0750 (18)	0.0733 (19)	0.0106 (14)	0.0013 (15)	0.0096 (14)
C2	0.0589 (16)	0.0502 (14)	0.0784 (19)	0.0069 (11)	−0.0127 (14)	0.0018 (13)
C3	0.087 (2)	0.0690 (18)	0.085 (2)	0.0123 (15)	−0.0181 (18)	−0.0046 (15)
C4	0.099 (3)	0.074 (2)	0.103 (3)	0.0135 (18)	−0.039 (2)	−0.0101 (19)
C5	0.0617 (19)	0.0684 (19)	0.147 (3)	0.0030 (14)	−0.036 (2)	−0.016 (2)
C6	0.0502 (16)	0.0723 (18)	0.120 (3)	−0.0008 (13)	−0.0081 (17)	−0.0153 (17)
C7	0.0466 (14)	0.0420 (13)	0.090 (2)	0.0037 (10)	−0.0076 (14)	−0.0032 (13)
C8	0.0431 (13)	0.0496 (14)	0.0794 (18)	0.0033 (10)	0.0015 (12)	−0.0006 (13)
C9	0.0587 (15)	0.0525 (14)	0.0719 (18)	−0.0067 (11)	0.0006 (13)	0.0012 (12)
C10	0.0579 (15)	0.0548 (15)	0.0735 (17)	−0.0081 (11)	0.0006 (13)	−0.0027 (13)
C11	0.0484 (14)	0.0508 (13)	0.0690 (17)	0.0086 (11)	0.0072 (12)	−0.0049 (12)
C12	0.0631 (17)	0.0680 (17)	0.081 (2)	−0.0004 (13)	0.0092 (15)	0.0131 (14)
C13	0.0562 (16)	0.0610 (16)	0.098 (2)	−0.0108 (12)	0.0009 (15)	0.0044 (15)
C14	0.0620 (15)	0.0597 (15)	0.0654 (17)	0.0130 (12)	0.0105 (13)	−0.0061 (12)
C15	0.0695 (15)	0.0446 (13)	0.0489 (14)	0.0077 (11)	0.0013 (12)	0.0008 (10)
C16	0.0570 (14)	0.0471 (13)	0.0448 (13)	0.0072 (10)	−0.0003 (11)	0.0001 (11)
C17	0.164 (3)	0.0500 (15)	0.0443 (15)	−0.0021 (17)	0.0204 (17)	−0.0055 (12)
C18	0.160 (3)	0.0517 (15)	0.0433 (15)	−0.0013 (17)	0.0203 (17)	0.0055 (12)
C19	0.0521 (13)	0.0433 (12)	0.0455 (13)	0.0053 (10)	0.0019 (10)	−0.0014 (10)
C20	0.095 (2)	0.0570 (15)	0.0540 (15)	−0.0191 (14)	0.0294 (14)	−0.0129 (12)
C21	0.0887 (19)	0.0570 (15)	0.0497 (15)	−0.0178 (13)	0.0238 (13)	−0.0036 (12)
C22	0.0552 (14)	0.0427 (12)	0.0398 (12)	0.0043 (10)	0.0065 (10)	0.0018 (10)
C23	0.0617 (16)	0.0504 (14)	0.0714 (17)	0.0070 (11)	−0.0006 (13)	0.0010 (12)
C24	0.0655 (16)	0.0589 (15)	0.0668 (17)	−0.0075 (12)	−0.0024 (13)	−0.0009 (13)
C25	0.0798 (18)	0.0471 (13)	0.0539 (15)	−0.0019 (13)	0.0085 (13)	−0.0008 (11)
C26	0.0755 (18)	0.0463 (14)	0.0756 (18)	0.0130 (12)	0.0075 (15)	0.0111 (12)
C27	0.0554 (14)	0.0511 (14)	0.0593 (15)	0.0034 (11)	−0.0005 (12)	0.0082 (11)
C28	0.072 (2)	0.0641 (17)	0.122 (3)	0.0005 (15)	−0.0199 (19)	0.0240 (17)
C29	0.0571 (14)	0.0423 (12)	0.0657 (16)	0.0014 (10)	0.0088 (12)	−0.0032 (11)
C30	0.0530 (13)	0.0381 (11)	0.0450 (12)	−0.0003 (9)	0.0009 (10)	−0.0029 (10)
C31	0.0697 (16)	0.0384 (12)	0.0647 (16)	0.0147 (11)	0.0157 (13)	0.0077 (11)
C32	0.0686 (16)	0.0444 (13)	0.0602 (15)	0.0089 (11)	0.0152 (12)	0.0098 (11)
C33	0.0513 (13)	0.0408 (12)	0.0432 (12)	0.0048 (9)	−0.0032 (10)	−0.0014 (10)
C34	0.0523 (13)	0.0342 (11)	0.0538 (14)	0.0033 (9)	−0.0034 (11)	0.0021 (10)
C35	0.0535 (13)	0.0385 (11)	0.0496 (13)	−0.0032 (10)	0.0018 (10)	0.0026 (10)
C36	0.0501 (13)	0.0379 (11)	0.0556 (14)	0.0018 (9)	0.0020 (11)	−0.0032 (10)
C37	0.0677 (16)	0.0667 (16)	0.0630 (17)	0.0152 (13)	0.0139 (13)	0.0042 (13)
C38	0.082 (2)	0.0767 (19)	0.082 (2)	0.0141 (16)	0.0280 (17)	0.0019 (15)
C39	0.0667 (18)	0.0627 (17)	0.097 (2)	0.0042 (13)	0.0250 (17)	−0.0140 (16)
C40	0.0468 (14)	0.0489 (14)	0.099 (2)	0.0018 (10)	−0.0033 (14)	−0.0088 (14)
C41	0.0529 (11)	0.0468 (10)	0.0684 (12)	0.0022 (9)	−0.0103 (11)	−0.0051 (10)
C42	0.0559 (11)	0.0533 (11)	0.0723 (13)	0.0054 (9)	−0.0128 (11)	−0.0034 (11)
N1	0.0548 (11)	0.0409 (10)	0.0530 (11)	0.0087 (8)	0.0009 (9)	−0.0067 (8)
N2	0.093 (2)	0.125 (2)	0.0771 (19)	0.0226 (15)	−0.0209 (15)	0.0079 (16)
N3	0.086 (2)	0.113 (2)	0.225 (4)	−0.0195 (17)	−0.059 (2)	0.054 (2)
N4	0.0671 (17)	0.121 (2)	0.094 (2)	0.0082 (15)	0.0118 (15)	0.0107 (16)

### Geometric parameters (Å, °)

**Table:**

C1—N4	1.138 (3)	C21—H21A	0.9300
C1—C2	1.432 (4)	C22—C23	1.381 (3)
C2—C3	1.386 (4)	C22—C27	1.399 (3)
C2—C7	1.406 (4)	C23—C24	1.381 (3)
C3—C4	1.363 (4)	C23—H23A	0.9300
C3—H3A	0.9300	C24—C25	1.364 (3)
C4—C5	1.368 (5)	C24—H24A	0.9300
C4—H4A	0.9300	C25—C26	1.365 (3)
C5—C6	1.390 (4)	C25—H25A	0.9300
C5—H5A	0.9300	C26—C27	1.384 (3)
C6—C7	1.399 (3)	C26—H26A	0.9300
C6—H6A	0.9300	C27—C28	1.424 (4)
C7—C8	1.485 (4)	C28—N3	1.139 (4)
C8—C13	1.382 (4)	C29—N1	1.463 (3)
C8—C9	1.394 (3)	C29—C30	1.504 (3)
C9—C10	1.369 (3)	C29—H29A	0.9700
C9—H9A	0.9300	C29—H29B	0.9700
C10—C11	1.383 (3)	C30—C35	1.380 (3)
C10—H10A	0.9300	C30—C31	1.380 (3)
C11—C12	1.379 (3)	C31—C32	1.379 (3)
C11—C14	1.501 (3)	C31—H31A	0.9300
C12—C13	1.379 (4)	C32—C33	1.394 (3)
C12—H12A	0.9300	C32—H32A	0.9300
C13—H13A	0.9300	C33—C34	1.388 (3)
C14—N1	1.473 (3)	C33—C36	1.480 (3)
C14—H14A	0.9700	C34—C35	1.376 (3)
C14—H14B	0.9700	C34—H34A	0.9300
C15—N1	1.468 (3)	C35—H35A	0.9300
C15—C16	1.505 (3)	C36—C37	1.380 (3)
C15—H15A	0.9700	C36—C41	1.394 (3)
C15—H15B	0.9700	C37—C38	1.377 (3)
C16—C21	1.365 (3)	C37—H37A	0.9300
C16—C17	1.374 (3)	C38—C39	1.364 (4)
C17—C18	1.379 (3)	C38—H38A	0.9300
C17—H17A	0.9300	C39—C40	1.358 (4)
C18—C19	1.373 (3)	C39—H39A	0.9300
C18—H18A	0.9300	C40—C41	1.405 (3)
C19—C20	1.361 (3)	C40—H40A	0.9300
C19—C22	1.490 (3)	C41—C42	1.429 (4)
C20—C21	1.380 (3)	C42—N2	1.156 (3)
C20—H20A	0.9300		
			
N4—C1—C2	179.0 (3)	C20—C21—H21A	119.5
C3—C2—C7	120.9 (3)	C23—C22—C27	116.7 (2)
C3—C2—C1	117.4 (3)	C23—C22—C19	121.39 (19)
C7—C2—C1	121.7 (2)	C27—C22—C19	121.9 (2)
C4—C3—C2	121.1 (3)	C24—C23—C22	121.9 (2)
C4—C3—H3A	119.5	C24—C23—H23A	119.0
C2—C3—H3A	119.5	C22—C23—H23A	119.0
C3—C4—C5	119.0 (3)	C25—C24—C23	120.1 (2)
C3—C4—H4A	120.5	C25—C24—H24A	119.9
C5—C4—H4A	120.5	C23—C24—H24A	119.9
C4—C5—C6	121.7 (3)	C24—C25—C26	119.8 (2)
C4—C5—H5A	119.2	C24—C25—H25A	120.1
C6—C5—H5A	119.2	C26—C25—H25A	120.1
C5—C6—C7	120.2 (3)	C25—C26—C27	120.3 (2)
C5—C6—H6A	119.9	C25—C26—H26A	119.9
C7—C6—H6A	119.9	C27—C26—H26A	119.9
C6—C7—C2	117.2 (3)	C26—C27—C22	121.1 (2)
C6—C7—C8	120.3 (3)	C26—C27—C28	119.1 (2)
C2—C7—C8	122.5 (2)	C22—C27—C28	119.7 (2)
C13—C8—C9	116.9 (2)	N3—C28—C27	177.1 (3)
C13—C8—C7	121.1 (2)	N1—C29—C30	113.78 (18)
C9—C8—C7	121.9 (2)	N1—C29—H29A	108.8
C10—C9—C8	121.1 (2)	C30—C29—H29A	108.8
C10—C9—H9A	119.5	N1—C29—H29B	108.8
C8—C9—H9A	119.5	C30—C29—H29B	108.8
C9—C10—C11	121.8 (2)	H29A—C29—H29B	107.7
C9—C10—H10A	119.1	C35—C30—C31	117.6 (2)
C11—C10—H10A	119.1	C35—C30—C29	120.0 (2)
C12—C11—C10	117.3 (2)	C31—C30—C29	122.31 (19)
C12—C11—C14	122.1 (2)	C32—C31—C30	121.8 (2)
C10—C11—C14	120.5 (2)	C32—C31—H31A	119.1
C13—C12—C11	121.0 (3)	C30—C31—H31A	119.1
C13—C12—H12A	119.5	C31—C32—C33	120.4 (2)
C11—C12—H12A	119.5	C31—C32—H32A	119.8
C12—C13—C8	121.8 (2)	C33—C32—H32A	119.8
C12—C13—H13A	119.1	C34—C33—C32	117.8 (2)
C8—C13—H13A	119.1	C34—C33—C36	122.11 (19)
N1—C14—C11	111.64 (19)	C32—C33—C36	120.1 (2)
N1—C14—H14A	109.3	C35—C34—C33	120.90 (19)
C11—C14—H14A	109.3	C35—C34—H34A	119.6
N1—C14—H14B	109.3	C33—C34—H34A	119.6
C11—C14—H14B	109.3	C34—C35—C30	121.6 (2)
H14A—C14—H14B	108.0	C34—C35—H35A	119.2
N1—C15—C16	112.73 (17)	C30—C35—H35A	119.2
N1—C15—H15A	109.0	C37—C36—C41	117.1 (2)
C16—C15—H15A	109.0	C37—C36—C33	119.8 (2)
N1—C15—H15B	109.0	C41—C36—C33	123.1 (2)
C16—C15—H15B	109.0	C38—C37—C36	121.8 (3)
H15A—C15—H15B	107.8	C38—C37—H37A	119.1
C21—C16—C17	117.1 (2)	C36—C37—H37A	119.1
C21—C16—C15	121.6 (2)	C39—C38—C37	120.4 (3)
C17—C16—C15	121.3 (2)	C39—C38—H38A	119.8
C16—C17—C18	121.8 (2)	C37—C38—H38A	119.8
C16—C17—H17A	119.1	C40—C39—C38	120.2 (3)
C18—C17—H17A	119.1	C40—C39—H39A	119.9
C19—C18—C17	120.6 (2)	C38—C39—H39A	119.9
C19—C18—H18A	119.7	C39—C40—C41	119.8 (2)
C17—C18—H18A	119.7	C39—C40—H40A	120.1
C20—C19—C18	117.5 (2)	C41—C40—H40A	120.1
C20—C19—C22	121.6 (2)	C36—C41—C40	120.8 (2)
C18—C19—C22	120.8 (2)	C36—C41—C42	120.2 (2)
C19—C20—C21	121.8 (2)	C40—C41—C42	118.9 (2)
C19—C20—H20A	119.1	N2—C42—C41	179.2 (3)
C21—C20—H20A	119.1	C29—N1—C15	111.04 (17)
C16—C21—C20	121.1 (2)	C29—N1—C14	109.79 (17)
C16—C21—H21A	119.5	C15—N1—C14	110.46 (18)

### Hydrogen-bond geometry (Å, °)

**Table:**

*D*—H···*A*	*D*—H	H···*A*	*D*···*A*	*D*—H···*A*
C4—H4A···N3^i^	0.93	2.55	3.446 (5)	162.
C24—H24A···N2^ii^	0.93	2.59	3.350 (4)	139.

Symmetry codes: (i) −*x*, *y*−1/2, −*z*+1/2; (ii) −*x*+1, −*y*+1, −*z*+2.
